# Influence of Polycarboxylate Superplasticizer on Rheological Behavior and Early Interfacial Evolution of Phosphogypsum-Based Supersulfated Cement

**DOI:** 10.3390/polym18091021

**Published:** 2026-04-23

**Authors:** Dafu Wang, Lehuan Kuang, Shaoyang Ding, Yudong Sun, Yuejing Li, Ziyu Chen, Jun Ren, Xincheng Li

**Affiliations:** 1 School of Architecture and Planning, Yunnan University, Kunming 650050, China; wangdafu92@163.com (D.W.); lehuank@163.com (L.K.); imdingdingking@163.com (S.D.); yudong.sun@outlook.com (Y.S.); 20251150288@stu.ynu.edu.cn (Y.L.); 20231150261@stu.ynu.edu.cn (Z.C.);; 2 Yunnan Key Laboratory of Carbon Neutrality and Green Low-Carbon Technologies, Yunnan University, Kunming 650050, China; 3 Yunnan Institute of Building Research, Kunming 650223, China; 4 Yunnan Key Laboratory of Building Structure and New Materials, Kunming 650223, China

**Keywords:** polycarboxylate superplasticizer, phosphogypsum, supersulfated cement, rheological properties, structural build-up, early interfacial evolution

## Abstract

Driven by global carbon reduction targets, supersulfated cement has emerged as a promising low-carbon cementitious material. This study investigates the influence of a polycarboxylate superplasticizer (PCE) on the rheological behavior and early interfacial evolution of phosphogypsum-based supersulfated cement (PSSC). Rheological measurements, pore solution ion analysis, hydration heat analysis, X-ray diffraction (XRD), and scanning electron microscopy coupled with energy-dispersive spectroscopy (SEM–EDS) are employed to correlate early hydration processes with structural development. The results indicate that the incorporation of PCE significantly reduces the initial yield stress and moderates the structural build-up rate. At a PCE dosage of 0.3 wt.%, the initial static yield stress decreases from 1313 Pa to approximately 125 Pa, while the structural build-up index *I_s_*_,*s*_ reaches 10.19, indicating improved particle dispersion while maintaining progressive structural reconstruction during hydration. Phosphogypsum (PG) functions not only as a sulfate source but also as an active interfacial substrate that promotes the preferential nucleation of AFt on its surface. In the absence of PCE, continuous Ca–P-enriched layers form on PG particles, accompanied by localized AFt accumulation. After the incorporation of PCE, the primary crystalline phases remain unchanged; however, gypsum dissolution and AFt formation are delayed. Meanwhile, Ca–P enrichment shifts from continuous coverage to a more dispersed distribution, promoting the spatially separated growth of AFt crystals rather than dense localized aggregation. Overall, PCE influences the evolution of the structure and properties of the system by regulating early interfacial reactions and the spatial organization of hydration products.

## 1. Introduction

Supersulfated cement (SSC) is a low-carbon cementitious material composed of 75–85% ground granulated blast furnace slag (GGBS), activated by 10–20% sulfate sources and a small fraction of an alkaline activator [1]. Owing to its extremely low clinker content, SSC provides a promising pathway for reducing the carbon footprint of the cement industry [2,3]. Phosphogypsum (PG), a by-product of the wet-process production of phosphoric acid [4,5,6], is generated in large quantities and commonly stockpiled, raising long-term environmental concerns [7,8,9]. Utilizing PG as a sulfate activator in SSC not only enables its large-scale recycling but also contributes to the development of low-carbon cementitious materials [10,11,12].

PG typically contains a high proportion of CaSO_4_·2H_2_O and exhibits a plate-like crystal morphology [13,14]. The accumulation of these crystals in fresh paste, together with the high specific surface area of GGBS particles, increases the water demand of the system and affects early structural build-up [15,16]. To improve workability, water-reducing agents such as calcium lignosulfonate, naphthalene-based superplasticizers, and polycarboxylate superplasticizers (PCE) have been widely applied [17,18,19,20,21]. Among them, PCE shows superior dispersion efficiency in gypsum-based binder systems because of its comb-like molecular architecture. In such structures, carboxyl groups adsorb onto particle surfaces, while the side chains provide steric hindrance, thereby mitigating particle flocculation and reducing water demand [22,23,24].

In phosphogypsum-based supersulfated cement (PSSC) systems, early hydration is strongly influenced by interfacial processes associated with PG particles [25]. The vicinity of gypsum phases can create localized ionic environments that influence early precipitation behavior [26]. Meanwhile, PG often contains soluble phosphorus and other impurities that interact with Ca^2+^ [27,28], which leads to the formation of Ca–P-enriched regions on particle surfaces [11,29]. Such interfacial enrichment has been reported to modify dissolution behavior and alter the local chemical environment, thereby influencing early reaction kinetics and structural build-up [30,31,32].

Recent work on PSSC has largely focused on bulk performance, with an emphasis on raw material pretreatment and chemical regulation. Liu [11] et al. reported that PG modification enhances hydration and strength development; in a separate study, the same group further demonstrated that lime neutralization alters impurity profiles and thereby affects fresh behavior, hydration products, and hardened properties [6]. Gracioli [33] et al. identified calcination temperature as a key variable controlling calcium sulfate availability and ettringite formation. In parallel, Xing [34] et al. highlighted the role of weak acid salts in governing hydration via pH adjustment, while Wu [35] et al. showed that soluble phosphorus can significantly prolong induction and setting by lowering pore-solution pH and promoting calcium phosphate-related precipitation. From an admixture perspective, Peng [20] et al. attributed the dispersing effect of polycarboxylate superplasticizers to their comb-like adsorption on gypsum surfaces, involving both steric and electrostatic interactions. Qi [36] et al. further reported that phosphate-modified PCEs improve water reduction but simultaneously suppress calcium sulfate dihydrate precipitation and delay setting. Accumulating evidence indicates that phosphorus species (H_3_PO_4_, H_2_PO_4_^−^, HPO_4_^2−^, and PO_4_^3−^) interfere with PCE adsorption and efficiency through the formation of phosphate-rich surface layers or insoluble calcium phosphate phases [37,38]. Similar dependencies have also been observed in slag-rich systems, where the performance of phosphate-based superplasticizers is strongly governed by binder chemistry, activator composition, and polymer structure [39].

However, existing studies have largely focused on bulk performance, with limited attention to early-stage interfacial processes and the coupling between phosphorus-containing environments and PCE. How these interactions govern ion transport, nucleation behavior, and the spatial distribution of hydration products in PSSC systems remains unclear. Accordingly, this study investigates the influence of PCE on the rheological behavior and early interfacial evolution of phosphogypsum-based supersulfated cement mortar (PSSCM). By integrating rheological testing, pore solution ion analysis, hydration heat measurement, and multi-scale microstructural characterization (XRD and SEM–EDS), the relationships between interfacial reactions, crystal spatial distribution, and macroscopic performance development are systematically explored.

## 2. Materials and Methods

### 2.1. Materials

**Figure 1:**
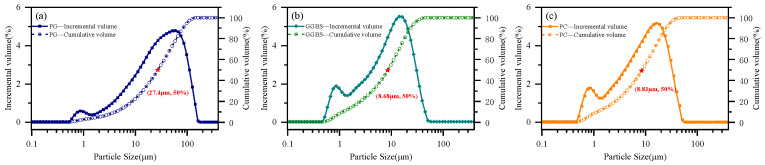
Particle size distributions of the raw materials: (**a**) phosphogypsum (PG); (**b**) ground granulated blast furnace slag (GGBS); (**c**) P·I 52.5 ordinary Portland cement (PC).

The raw materials included Phosphogypsum PG, ground granulated blast furnace slag (GGBS), P·I 52.5 ordinary Portland cement (PC), polycarboxylate superplasticizer (PCE), and standard sand. The washed PG was supplied by Yunnan Xiangfeng Industrial Group Co., Ltd. (Anning, China), with a median particle size (d_50_) of 27.4 μm. The PC, produced by Qingdao Shanshui Innovation Cement Co., Ltd. (Qingdao, China), had a density of 3.18 g/cm^3^, a specific surface area of 310 m^2^/kg, and a d_50_ of 8.82 μm. The S95-grade GGBS was obtained from Qingdao Runyi Fengtai New Materials Technology Co., Ltd. (Qingdao, China), with a density of 2.88 g/cm^3^, a specific surface area of 415 m^2^/kg, and a d_50_ of 8.68 μm. These particle size parameters were derived from the particle size distributions shown in Figure 1. The ether-type PCE was supplied by Sichuan Dongrun Baisheng New Materials Co., Ltd. (Chengdu, China), with a solid content of 20 wt.% and a water-reducing efficiency of 27%. Chinese ISO standard sand was provided by Xiamen ISO Standard Sand Co., Ltd. (Xiamen, China).

**Figure 2:**
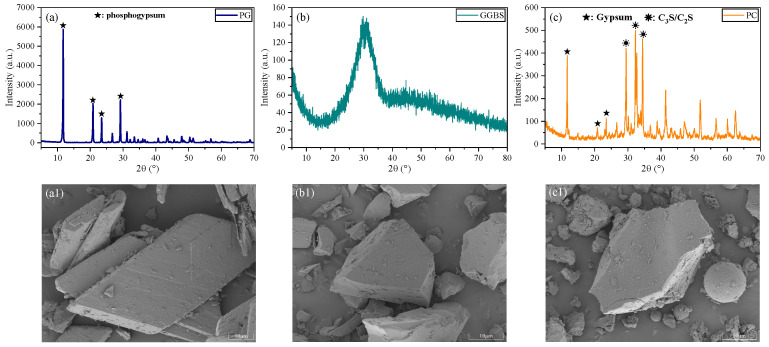
Phase compositions and microstructural characteristics of raw materials: (**a**) XRD pattern of PG; (**b**) XRD pattern of GGBS; (**c**) XRD pattern of PC; (**a1**) SEM image of PG; (**b1**) SEM image of GGBS; (**c1**) SEM image of PC.

The particle size distributions and chemical compositions of PG, GGBS, and PC are shown in Figure 1 and Table 1, respectively. As shown in Figure 1, PG exhibits a relatively coarser and broader particle size distribution, whereas GGBS and PC exhibit finer and more concentrated distributions. The microstructures and phase compositions of PG, GGBS, and PC are presented in Figure 2. As shown in Figure 2, PG was mainly composed of CaSO_4_·2H_2_O and exhibited a plate-like morphology with relatively smooth surfaces, while GGBS displayed a predominantly amorphous structure characterized by a broad diffraction hump and irregular particle shapes. In contrast, PC showed distinct crystalline phases such as C_3_S and C_2_S, accompanied by a comparatively dense particle morphology. These differences in particle size are expected to influence particle packing and dispersion behavior and may further affect early hydration processes in the PSSC system.

### 2.2. Experimental Design

The experimental program consisted of two sequential phases. The first phase evaluated the influence of PCE dosage on the rheological behavior of phosphogypsum-based supersulfated cement mortar (PSSCM). Mixtures containing 0–0.8 wt.% PCE (by mass of cementitious materials) were prepared to assess early yield stress and structural build-up, while the water-to-binder ratio (W/B) was kept constant at 0.4 for all mixtures to isolate the intrinsic effect of PCE on particle dispersion and rheological evolution. This approach avoids the influence of varying water content and ensures that differences in rheological behavior are primarily attributed to PCE rather than to changes in mixture composition.

Based on the rheological results, a representative PCE dosage was selected for subsequent analysis. The second phase focused on early hydration characteristics and interfacial evolution, including pore solution ion analysis, hydration heat measurements, XRD, and SEM–EDS characterization. The detailed mix proportions are listed in Table 2.

### 2.3. Sample Preparation

**Figure 3:**
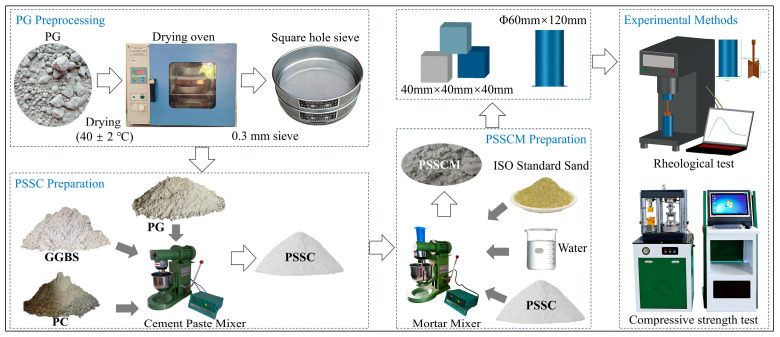
Schematic illustration of raw material pretreatment, Phosphogypsum-based supersulfated cement (PSSC) and phosphogypsum-based supersulfated cement mortar (PSSCM) preparation, and performance testing. The arrows indicate the process flow.

Prior to mixing, PG was dried at (40 ± 2) °C to constant mass to remove residual moisture and then sieved through a 600-mesh sieve. The preparation procedure is illustrated in Figure 3. PG, GGBS, and PC were weighed according to the designed proportions and dry-blended for 2 min in a paste mixer.

PSSCM was prepared using a mortar mixer (JJ-5, Wuxi Jianyi Instrument Machinery Co., Ltd., Wuxi, China). Water and PCE were premixed before the addition of the dry binder. The mixture was stirred at low speed for 30 s, followed by the incorporation of standard sand. Subsequently, mixing was performed at high speed for 60 s, paused for 90 s, and then continued at high speed for an additional 60 s. The ambient temperature during mixing and testing was maintained at (20 ± 3) °C.

### 2.4. Test Methods

#### 2.4.1. Rheological Test

The early rheological properties of PSSCM were measured using a TR-MRI mortar rheometer (Shanghai Tongrui Instrument Equipment Co., Ltd., Shanghai, China) at hydration times of 4, 20, 40, 60, 80, 100, 120, 140, 160, and 180 min after mixing. Each sampling procedure was completed within 2 min. The rheometer operates with a programmable rotational speed ranging from 0.01 to 3000 rpm (minimum shear rate of 0.1 s^−1^).

**Figure 4:**
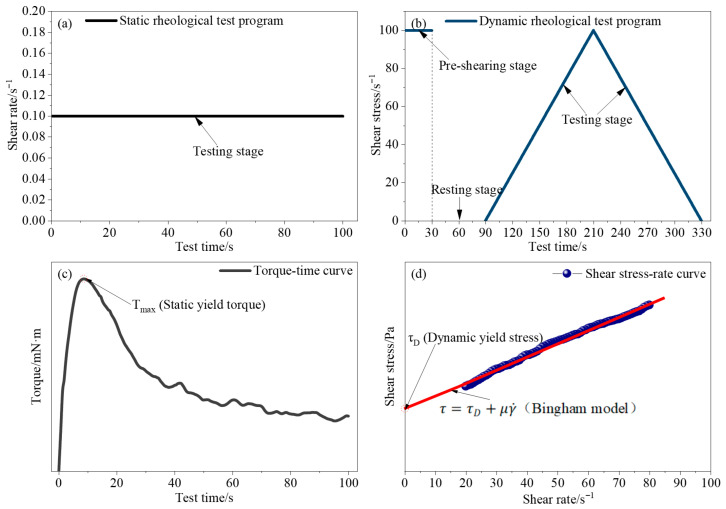
Rheological testing protocols and parameter determination: (**a**) static shear protocol; (**b**) dynamic up–down shear protocol; (**c**) determination of static yield stress ($\tau_{s}$) from the torque–time curve; (**d**) Bingham model fitting of the descending shear stress–shear rate curve to determine dynamic yield stress ($\tau_{D}$) and plastic viscosity (μ).

The static yield stress ($\tau_{s}$) was determined from the peak torque in the torque–time curve under a constant rotational speed of 0.6 rpm, as illustrated in Figure 4a,c and according to Equation (1). The dynamic yield stress ($\tau_{D}$) and plastic viscosity (μ) were obtained using an up–down shear protocol (Figure 4b,d). Specifically, samples were pre-sheared at 100 s^−1^ for 30 s, followed by a resting period of 60 s, after which the shear rate was linearly increased from 0 to 100 s^−1^ over 120 s and then decreased back to 0 s^−1^ over 120 s. Data were recorded at 1 s intervals. The Bingham model was applied to the descending branch of the shear stress–shear rate curve between 20 and 80 s^−1^ to determine $\tau_{D}$ and μ (Figure 4d). Key stages of the rheological protocol (pre-shearing, resting, and testing), as well as the determination of $\tau_{s}$, $\tau_{D}$, and the Bingham model fit, are clearly annotated in Figure 4 to facilitate interpretation and ensure reproducibility.

(1) $$\tau_{s}=\frac{2T_{m}}{\pi D^{3}\left(\frac{H}{D}-\frac{1}{3}\right)}$$

To quantify structural evolution, the structural build-up index (*I*_s_) was defined as follows:

(2) $$I_{s}=\frac{\tau_{180\min}-\tau_{4\min}}{\tau_{4\min}}$$

where *τ* represents the yield stress at the corresponding hydration time. This parameter reflects the relative increase in yield stress within the testing interval.

#### 2.4.2. Hydration Heat Analysis

The hydration heat evolution of (Phosphogypsum-based supersulfated cement) PSSC was measured using a TAM Air eight-channel isothermal calorimeter. Heat flow and cumulative heat release were continuously recorded for 72 h at a controlled temperature of (20 ± 0.1) °C. The mass of each sample was maintained at (5.0 ± 0.1) g.

#### 2.4.3. Ion Concentration Analysis

The concentrations of Al (OH)_4_^−^, Ca^2+^, SO_4_^2−^, and Na^+^ in the pore solution were quantified using an Agilent 720ES inductively coupled plasma optical emission spectrometer (ICP-OES, Agilent Technologies, Santa Clara, CA, USA). Samples were extracted at hydration times of 4–180 min, centrifuged at 4000 rpm for 10 min, and filtered through a 0.22 μm membrane filter. The filtrate was acidified with 0.5 vol% HNO_3_ and diluted at a ratio of 1:10 prior to analysis [40].

#### 2.4.4. Microstructural Characterization (SEM/EDS/XRD)

Microstructural features were characterized by scanning electron microscopy (SEM, Hitachi SU8010, Hitachi High-Tech Corporation, Tokyo, Japan) coupled with energy-dispersive spectroscopy (EDS), and phase composition was determined by X-ray diffraction (XRD, Bruker D8 Advance, Bruker AXS GmbH, Karlsruhe, Germany) with a scanning rate of 2°/min. Hydration was stopped at designated times by immersion in anhydrous ethanol, followed by filtration and drying at 40–50 °C prior to analysis.

#### 2.4.5. Compressive Strength Test

Mortar specimens (40 mm × 40 mm × 40 mm) were cast and cured under standard conditions. Compressive strength was measured at 3, 7, and 28 days using a universal testing machine under uniaxial loading at a rate of 2.4 kN/s until failure. Three replicate specimens were tested for each mixture, and the average value was reported.

## 3. Results and Discussion

### 3.1. Effect of PCE Dosage on Rheological Behavior

Rheological behavior reflects the evolution of the particle network and provides insight into the early structural build-up in PSSCM. Since PCE primarily functions as a dispersing agent, variations in its dosage are expected to influence the formation and reconstruction of the particle network. Therefore, the evolution of yield stress and viscosity at different PCE dosages is first examined.

#### 3.1.1. Evolution of Yield Stress

**Figure 5:**
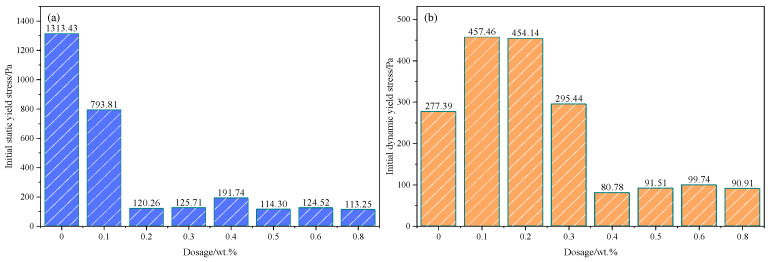
Initial yield stress of PSSCM at different PCE dosages: (**a**) $\tau_{S}$; (**b**) $\tau_{D}$.

The initial yield stress of PSSCM at different PCE dosages is summarized in Figure 5. The control mixture (PCE-0.0) shows a relatively high initial $\tau_{S}$ of 1313 Pa, indicating the rapid formation of a flocculated particle network immediately after mixing. With the incorporation of 0.1 wt.% PCE, the initial $\tau_{S}$ decreases markedly to 793 Pa, suggesting effective particle dispersion in the fresh mixture. A similar trend is observed for $\tau_{D}$. Compared with the control mixture (277 Pa), $\tau_{D}$ increases to approximately 457 Pa at low PCE dosages.

At intermediate dosages (0.2–0.3 wt.%), both $\tau_{S}$ and $\tau_{D}$ are further reduced. The initial $\tau_{S}$ decreases to approximately 120–126 Pa, while $\tau_{D}$ remains around 450 Pa, indicating that PCE significantly weakens interparticle attraction and enhances particle dispersion. When the PCE dosage exceeds 0.4 wt.%, the initial yield stresses remain at relatively low levels with only minor variations, suggesting that the dispersion effect approaches saturation. This phenomenon can be attributed to the adsorption of PCE molecules on the surface of cementitious particles, which introduces steric hindrance and electrostatic repulsion, thereby reducing interparticle attraction and improving flowability, consistent with previous studies in cementitious systems [41].

**Figure 6:**
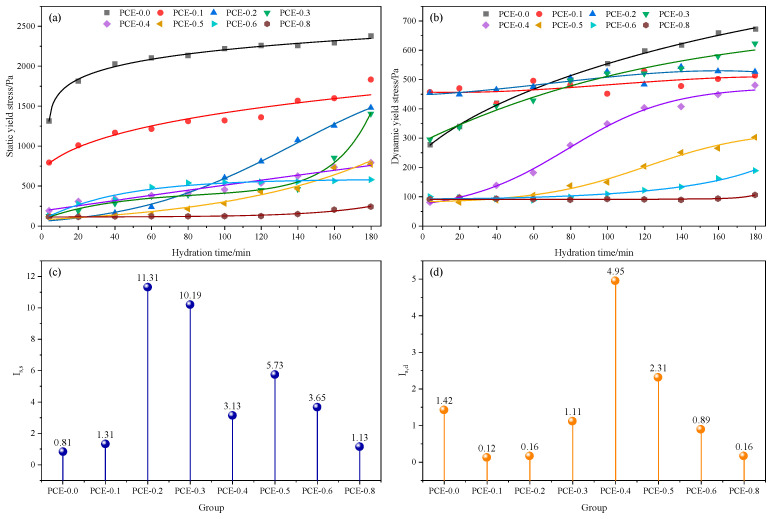
Rheological evolution of PSSCM with varying PCE dosages: (**a**) $\tau_{S}$ as a function of hydration time; (**b**) $\tau_{D}$ as a function of hydration time; (**c**) structural build-up indices $I_{S,S}$; (**d**) structural build-up indices $I_{S,D}$.

The temporal evolution of $\tau_{S}$ and $\tau_{D}$ during hydration is presented in Figure 6a and Figure 6b. In the control mixture, both parameters increase continuously with time. As shown in Figure 6a, $\tau_{S}$ rises from about 1313 Pa to approximately 2350 Pa, while $\tau_{D}$ increases from 277 Pa to nearly 670 Pa in Figure 6b at 180 min. Such behavior reflects the rapid formation of a flocculated particle network and the gradual development of structural rigidity in the absence of PCE. With the addition of PCE, the initial yield stresses are substantially reduced, and their subsequent evolution during hydration becomes strongly dependent on the PCE dosage.

The evolution of $\tau_{S}$ varies markedly with PCE dosage. As illustrated in Figure 6a, mixtures containing 0.2–0.3 wt.% PCE exhibit the most pronounced increase in $\tau_{S}$ during hydration. In these systems, $\tau_{S}$ increases from approximately 120–126 Pa to about 1400–1450 Pa, corresponding to the highest structural build-up indices $I_{S,S}$ (11.31 and 10.19), as shown in Figure 6c. Despite the low initial yield stress, the particle network progressively reforms as hydration proceeds. The increase in $\tau_{S}$ therefore follows a nonlinear trend with time, which can be reasonably approximated by a logarithmic-type growth. When the PCE dosage exceeds 0.4 wt.%, the increase in $\tau_{S}$ becomes less pronounced, and the $I_{S,S}$ values decrease accordingly (Figure 6c), indicating that excessive dispersion limits the recovery of the static particle structure.

The evolution of $\tau_{D}$ shows a different trend. As presented in Figure 6b, $\tau_{D}$ remains nearly constant at 0.1–0.2 wt.% PCE, leading to very small $I_{S,D}$ values (0.12–0.16) in Figure 6d. At 0.4 wt.% PCE, however, $\tau_{D}$ increases markedly from about 81 Pa to roughly 450 Pa, producing the highest $I_{S,D}$ (4.95). This behavior indicates a stronger recovery of shear resistance as hydration proceeds. With further increases in PCE dosage, the growth of $\tau_{D}$ decreases again, suggesting that strong and persistent particle dispersion inhibits the reconstruction of the shear-induced particle network. Overall, the results show that the PCE dosage favorable for static structural build-up (0.2–0.3 wt.%) differs from that promoting dynamic structural recovery (around 0.4 wt.%).

#### 3.1.2. Viscosity Evolution

**Figure 7:**
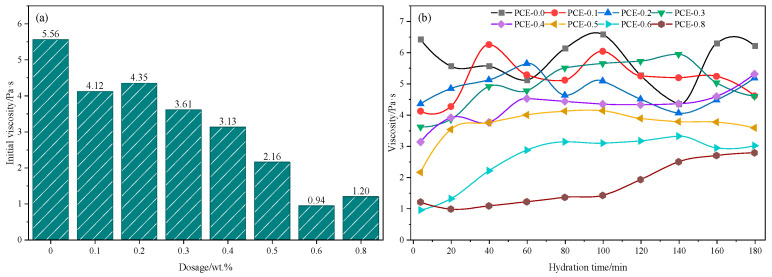
Evolution of μ of PSSCM with varying PCE dosages: (**a**) initial μ; (**b**) μ versus hydration time.

The evolution of μ under different PCE dosages is shown in Figure 7. As presented in Figure 7a, the control mixture (PCE-0.0) shows the highest initial viscosity of 5.56 Pa·s, reflecting strong interparticle interactions and the rapid formation of a flocculated particle network after mixing. With the addition of PCE, the initial μ progressively decreases, indicating enhanced particle dispersion and reduced interparticle resistance. At intermediate dosages (0.2–0.3 wt.% PCE), the initial μ decreases to approximately 4.35–3.61 Pa·s. As shown in Figure 7b, the viscosity in this dosage range remains relatively stable during hydration, suggesting that PCE weakens particle flocculation while still allowing gradual structural evolution of the suspension. When the PCE dosage exceeds 0.4 wt.%, the initial μ further decreases to approximately 3.13–0.94 Pa·s, and the viscosity remains at relatively low levels throughout the testing period. However, the additional reduction becomes less pronounced at higher dosages, indicating that the dispersion effect of PCE gradually approaches saturation.

Considering the combined evolution of $\tau_{S}$, $\tau_{D}$, and *μ*, the mixture containing 0.3 wt.% PCE shows reduced initial flow resistance while maintaining progressive structural build-up during hydration. Therefore, 0.3 wt.% PCE is selected as the representative dosage for subsequent analysis.

### 3.2. Hydration Heat Evolution

**Figure 8:**
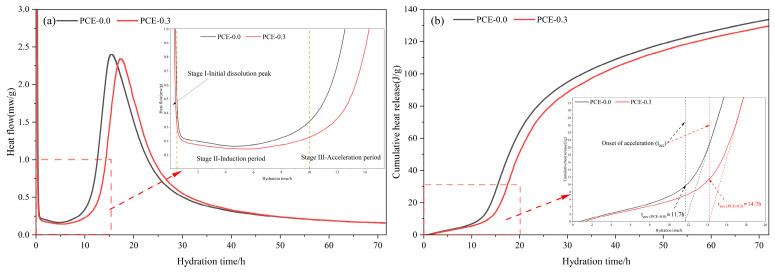
Hydration heat evolution of PSSC with and without PCE: (**a**) heat flow curves showing the dissolution peak, induction period, and acceleration stage; (**b**) cumulative heat release curves, with the onset of the acceleration stage (t_acc_) indicated in the inset based on the slope change.

To clarify whether the modified rheological behavior is accompanied by corresponding changes in early reaction kinetics, the hydration heat evolution of PSSCM with and without PCE is examined (Figure 8). The heat flow curves in Figure 8a show the typical early hydration stages, including Stage I (initial dissolution peak), Stage II (induction period), and Stage III (acceleration period).

As shown in Figure 8a, the incorporation of 0.3 wt.% PCE slightly delays the transition from the induction stage to the acceleration stage, resulting in a later main exothermic peak compared with the control mixture. Meanwhile, the peak intensity becomes slightly lower, indicating a reduction in the maximum reaction rate. During the induction stage (Stage II), the heat flow of the PCE-0.3 mixture remains consistently lower than that of the control mixture, suggesting that the adsorption of PCE on particle surfaces delays early nucleation and slows the formation of initial hydration products. During Stage III (acceleration period), both mixtures exhibit a pronounced heat release peak, associated with rapid hydration and the development of the particle network. However, the peak of the PCE-containing mixture appears later and with reduced magnitude, indicating that PCE moderates the early hydration kinetics and redistributes heat release over a longer period rather than suppressing the reaction entirely.

The cumulative heat release curves shown in Figure 8b further support this interpretation. To quantitatively characterize the transition from the induction period to the acceleration stage, a characteristic time parameter (t_acc_) is introduced, representing the onset of the acceleration stage. This parameter is determined from the change in slope of the cumulative heat release curves using a tangent method, as illustrated in the inset of Figure 8b. Based on this definition, t_acc_ is approximately 11.7 h for the PCE-free system and 14.2 h for the PCE-containing system. These results indicate that the incorporation of PCE delays the onset of the acceleration stage. Although the cumulative heat release of the PCE-0.3 mixture remains slightly lower during the early period, it exhibits a delayed but more gradual increase. The overall difference gradually diminishes at later times, suggesting that PCE primarily affects the temporal distribution of hydration heat, while the overall extent of hydration remains largely unchanged.

Calorimetric results show that the addition of PCE prolongs the induction period and delays the onset of the acceleration stage (t_acc_), as indicated by the shift in the cumulative heat release curves. This retardation effect of PCE on hydration kinetics has been widely observed and is commonly attributed to the dispersing action of PCE molecules, which reduces effective contact between cement particles and water, and to PCE interactions with Ca^2+^ and aluminate species that alter initial dissolution rates [42]. Calorimetric measurements reflect the integrated heat evolution of the system and cannot directly distinguish individual reaction pathways.

### 3.3. Pore Solution Ion Evolution

**Figure 9:**
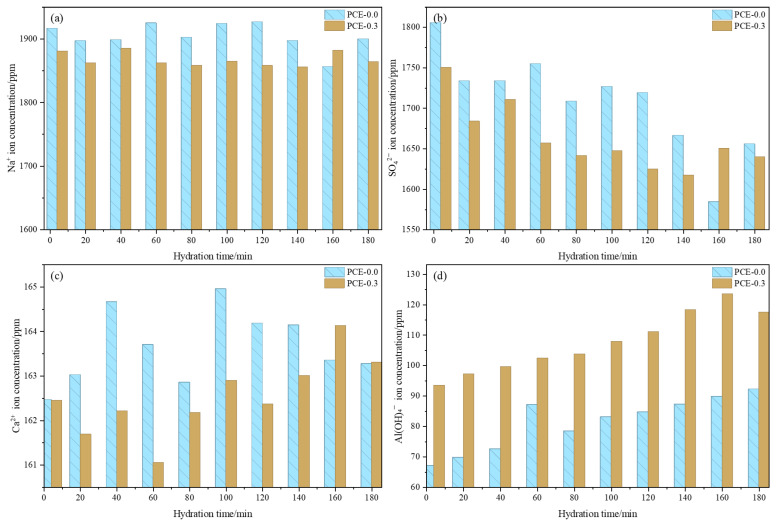
Evolution of major ion concentrations in the pore solution of PSSCM with and without PCE: (**a**) Na^+^; (**b**) SO_4_^2−^; (**c**) Ca^2+^; (**d**) Al (OH)_4_^−^.

To further clarify the origin of the moderated hydration kinetics observed in Section 3.2, the temporal evolution of major ion concentrations in the pore solution is examined (Figure 9). As shown in Figure 9a, the Na^+^ concentration remains within a relatively stable range in both mixtures throughout hydration, and no significant difference is observed between the control and PCE-0.3 mixture, indicating that the addition of PCE does not significantly alter the alkalinity of the pore solution. In contrast, the concentration of SO_4_^2−^ gradually decreases with hydration time in both mixtures (Figure 9b), reflecting its continuous participation in early reactions. The PCE-0.3 mixture exhibits slightly lower SO_4_^2−^ concentrations during the early stage, although the overall decreasing trend remains similar to that of the control mixture. This reduction in sulfate concentration does not necessarily indicate an accelerated overall reaction rate; rather, it suggests continuous sulfate consumption during AFt formation under a modified nucleation environment.

More noticeable differences appear for Ca^2+^ and Al(OH)_4_^−^. As shown in Figure 9c, the Ca^2+^ concentration in the PCE-0.3 mixture remains slightly lower and shows delayed fluctuations compared with the control mixture, indicating moderated release and redistribution of Ca^2+^ in the pore solution. Meanwhile, the concentration of Al(OH)_4_^−^ remains consistently higher in the PCE-containing mixture (Figure 9d), suggesting a delayed precipitation of Al-bearing hydration products. This behavior indicates that, although the reactant ions required for AFt formation are sufficiently available, their consumption through precipitation is postponed, reflecting a delay in effective nucleation rather than a limitation in ion supply.

Overall, the presence of PCE slightly delays the evolution of several key ionic species, which is consistent with the moderated hydration kinetics and delayed structural build-up discussed in the previous sections. More importantly, PCE alters the spatial characteristics of early hydration reactions by shifting AFt nucleation from localized growth on PG surfaces to a more dispersed mode within the pore solution. As a result, sulfate and calcium ions are continuously consumed, leading to relatively lower SO_4_^2−^ concentrations, while the formation of a continuous particle network is delayed due to the reduced connectivity of hydration products. In summary, the presence of PCE prolongs the residence time of key ionic species in the pore solution and primarily influences the temporal progression of early reactions rather than the overall extent of reaction. This suggests that early hydration behavior is governed not only by reaction kinetics but also by the spatial distribution and connectivity of hydration products. It should be noted that pore solution analysis reflects only the instantaneous chemical state of the system and cannot directly identify specific reaction pathways; therefore, further insight requires microstructural characterization.

### 3.4. Microstructural Evolution and Interfacial Characteristics

To clarify the microstructural basis underlying the observed kinetic and rheological differences, SEM/EDS and XRD analyses were performed, focusing on the spatial distribution of hydration products and the interfacial characteristics of PG particles in mixtures with and without the incorporation of PCE.

#### 3.4.1. Heterogeneous Nucleation of AFt on PG Surfaces

**Figure 10:**
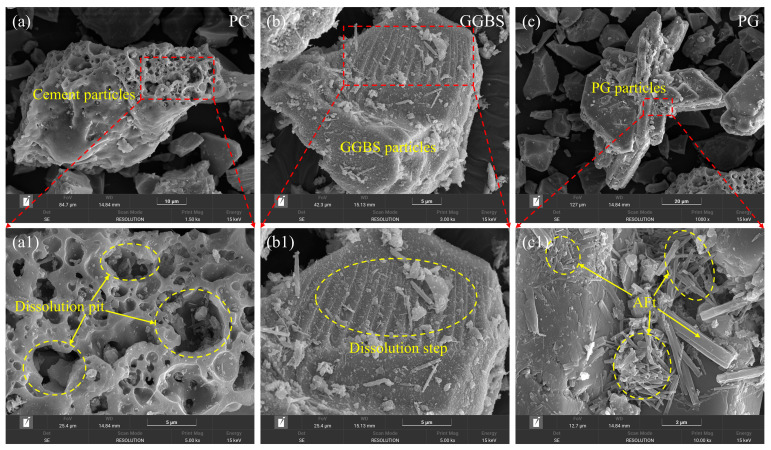
Early surface morphology of individual raw material particles in PSSC: (**a**) SEM image of PC; (**b**) SEM image of GGBS; (**c**) SEM image of PG; (**a1**) enlarged view of PC; (**b1**) enlarged view of GGBS; (**c1**) enlarged view of PG.

SEM observations of individual PC, GGBS, and PG particles at early hydration ages are shown in Figure 10. As shown in Figure 10a,a1, PC particles exhibit numerous dissolution pits, indicating rapid surface dissolution during early hydration. In contrast, GGBS particles (Figure 10b,b1) remain relatively dense, with only localized dissolution steps observed on their surfaces. PG particles display a markedly different morphology. As illustrated in Figure 10c,c1, needle-like and columnar crystals are directly attached to the PG surfaces. These crystals, morphologically identified as AFt, are preferentially distributed along the PG interface and locally form aggregated clusters.

Compared with PC and GGBS particles, whose surface morphologies are dominated by dissolution features, PG particles display clear evidence of secondary crystal precipitation. The direct attachment and alignment of AFt crystals along PG surfaces suggest that PG acts as a heterogeneous nucleation substrate during early hydration, thereby promoting the localized formation of AFt at the particle–solution interface.

#### 3.4.2. Influence of PCE on AFt Growth and Spatial Organization

**Figure 11:**
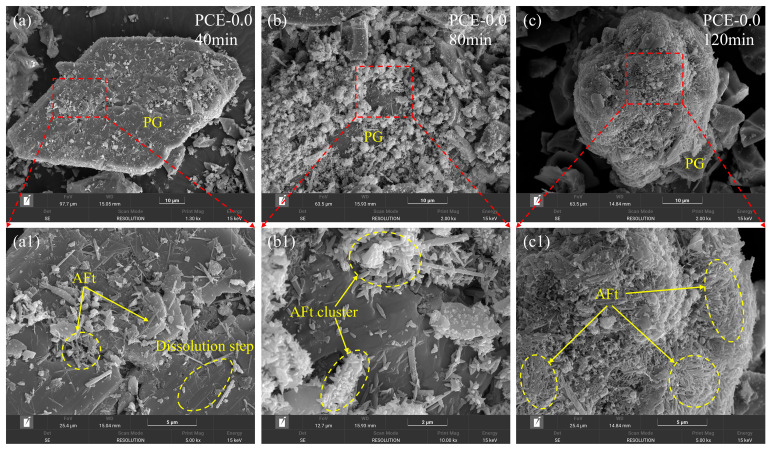
SEM images showing the evolution of AFt nucleation and growth on PG particle surfaces in the PCE-0.0 mixture at different hydration times. (**a**–**c**) Low-magnification images at 40, 80, and 120 min; (**a1**–**c1**) corresponding high-magnification views highlighting AFt precipitation, cluster formation, and dissolution-step features on the particle surfaces.

In the PCE-0.0, the temporal evolution of AFt morphology is shown in Figure 11. AFt exhibits pronounced localized growth during early hydration. At 40 min (Figure 11a,a1), needle-like crystals appear in confined interfacial regions on PG surfaces. With increasing hydration time, these crystals rapidly accumulate and form dense clusters at 80 min (Figure 11b,b1). By 120 min (Figure 11c,c1), the crystals further extend and interweave, producing locally compact covering layers on the particle surfaces. This growth pattern indicates a spatially concentrated precipitation process, characterized by the rapid formation of high-density AFt aggregates.

**Figure 12:**
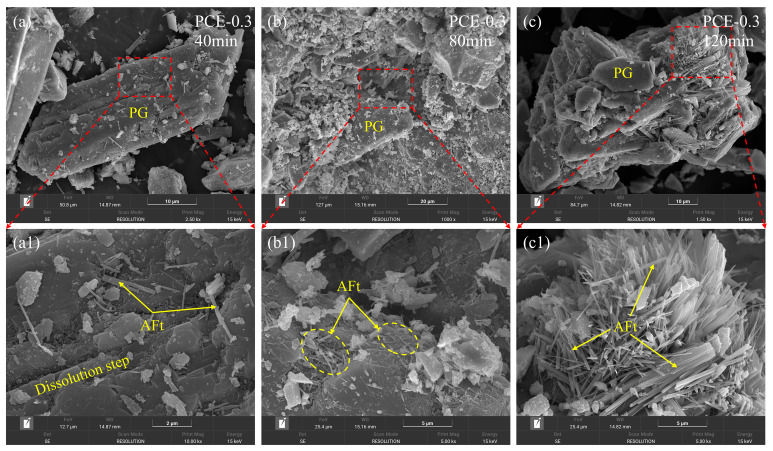
SEM images showing the evolution of AFt nucleation and growth on PG particle surfaces in the PCE-0.3 mixture at different hydration times. (**a**–**c**) Low-magnification images at 40, 80, and 120 min; (**a1**–**c1**) corresponding high-magnification views illustrating AFt morphology, spatial distribution, and dissolution-step features on the particle surfaces.

In contrast, the evolution of AFt morphology in the PCE-0.3 mixture is presented in Figure 12. At 40 min (Figure 12a,a1), fewer AFt crystals are observed, and their distribution remains relatively dispersed. At 80 min (Figure 12b,b1), the crystals mainly appear as slender needles, without forming dense localized aggregates. Even at 120 min (Figure 12c,c1), although radial crystal growth is evident, the overall structure remains relatively open, and no continuous compact layer forms on the particle surfaces. As a result, the spatial organization of AFt evolves in a more dispersed and progressive manner compared with the control mixture.

Based on the SEM observations, the incorporation of PCE modifies the early dissolution–reprecipitation behavior at the PG interface. This regulation of interfacial processes promotes a more spatially distributed development of AFt, thereby delaying the formation of compact interfacial frameworks. The observed morphological evolution is consistent with the reduced yield stress and delayed structural build-up reported in Section 3.1, as well as the moderated hydration heat evolution discussed in Section 3.2. These results suggest that PCE primarily regulates the spatial organization and growth dynamics of AFt during early hydration, rather than fundamentally altering the overall hydration pathway of the system.

#### 3.4.3. Phase Evolution of Hydration Products (XRD)

**Figure 13:**
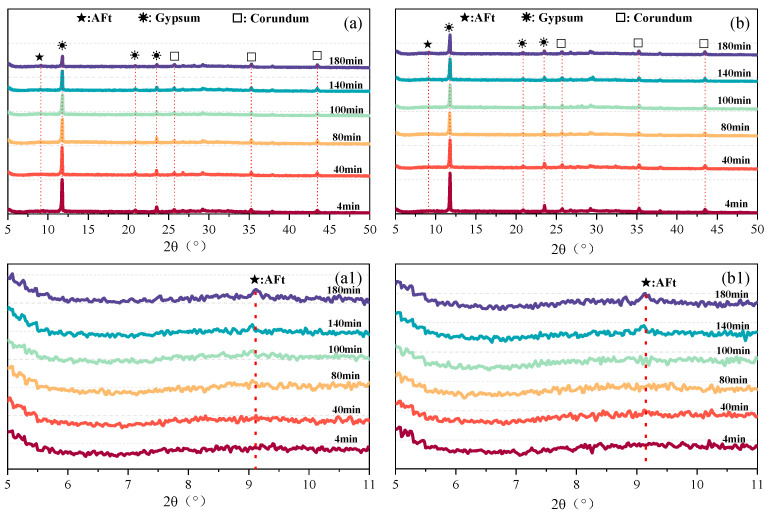
XRD patterns of the PCE-0.0 and PCE-0.3 mixtures at different hydration times: (**a**) full diffraction patterns of the PCE-0.0 mixture; (**b**) full diffraction patterns of the PCE-0.3 mixture; (**a1**) enlarged view of the characteristic AFt region for the PCE-0.0 mixture; (**b1**) enlarged view of the characteristic AFt region for the PCE-0.3 mixture.

The XRD patterns of the PCE-0.0 and PCE-0.3 mixtures at different hydration times are presented in Figure 13. As shown in the full diffraction patterns (Figure 13a,b), gypsum and AFt remain the dominant crystalline phases throughout the early hydration period (4–180 min), and no additional crystalline phases are detected in either mixture, indicating that the incorporation of PCE does not modify the primary phase assemblage of the system. The gypsum peaks mainly originate from the residual crystalline phase of the PG raw material; therefore, the gradual decrease in peak intensity reflects the progressive dissolution and consumption of PG. Compared with the control mixture, the attenuation of gypsum peaks in the PCE-0.3 mixture appears slightly slower during the early stage (Figure 13b1), suggesting that the participation of gypsum in the reaction process is moderately delayed.

A magnified view of the characteristic AFt diffraction region (2θ ≈ 9.1°, Figure 13a1,b1) further reveals differences in AFt crystallization behavior. In the PCE-0.3 mixture, the AFt peak remains relatively weak at early ages (≤40 min) and becomes progressively more pronounced with increasing hydration time (≥100 min), indicating a gradual development of AFt crystallinity and a slight delay in the appearance of detectable AFt.

**Figure 14:**
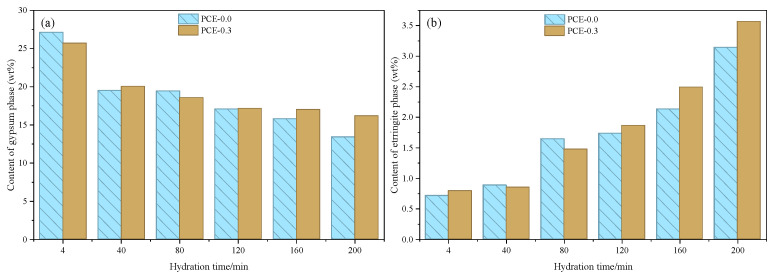
Semi-quantitative phase evolution determined by the internal standard method (20 wt.% corundum). (**a**) content of gypsum phase as a function of hydration time; (**b**) content of ettringite phase as a function of hydration time.

Semi-quantitative analysis based on the internal standard method (Figure 14) further supports this phase evolution. As shown in Figure 14a, the gypsum content decreases progressively with hydration time in both mixtures, dropping from about 26–27 wt.% at 4 min to approximately 13–16 wt.% at 200 min, reflecting the continuous dissolution of PG. Meanwhile, the AFt content increases correspondingly (Figure 14b), rising from about 0.7–0.8 wt.% at 4 min to roughly 3.1–3.5 wt.% at 200 min, indicating ongoing AFt formation during early hydration. Although slight differences are observed between the two mixtures, the overall AFt contents remain comparable, suggesting that PCE primarily modifies the rate and timing of AFt formation rather than its final amount. This behavior is consistent with the moderated hydration kinetics and delayed structural evolution discussed in the previous sections.

#### 3.4.4. Ca–P Enrichment Behavior and Spatial Distribution on PG Surfaces

**Figure 15:**
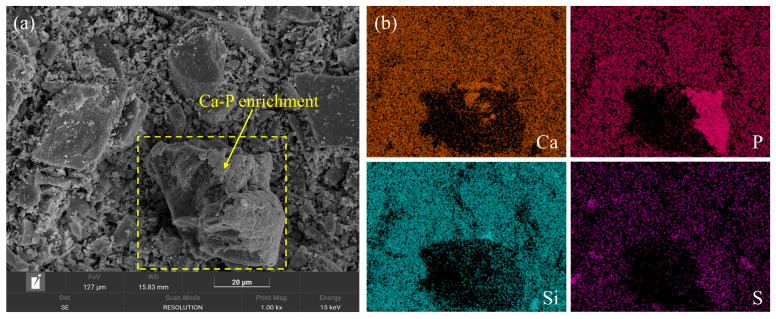
SEM image and corresponding EDS elemental maps of a PSSC particle: (**a**) SEM image showing Ca–P-enriched regions; (**b**) EDS elemental maps showing the distributions of Ca, P, Si, and S.

As shown in Figure 15, distinct Ca–P-enriched regions are observed on the surfaces of PG particles during early hydration. Elemental mapping reveals that Ca and P are strongly concentrated in localized areas, whereas elements such as Si and S are relatively uniformly distributed. This enrichment suggests that phosphorus-containing impurities associated with PG tend to accumulate together with Ca near the particle surfaces, forming Ca–P-rich regions. The formation of these enriched domains indicates that PG surfaces provide a chemically heterogeneous interfacial environment, which may influence both the dissolution behavior of PG and the nucleation of early hydration products.

**Figure 16:**
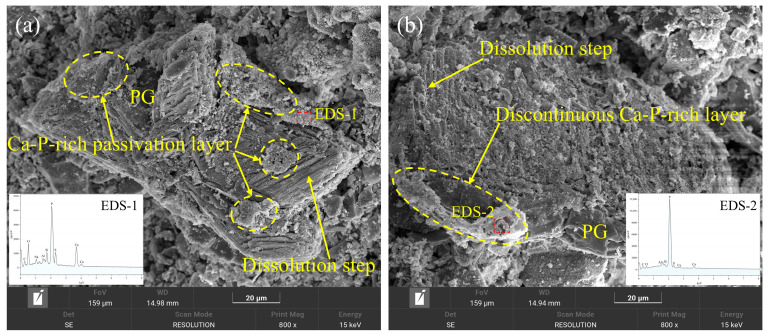
SEM–EDS characterization of Ca–P-enriched regions on PG particle surfaces: (**a**) PCE-0.0 showing a continuous Ca–P-enriched surface layer; (**b**) PCE-0.3 mixture showing discontinuous Ca–P-enriched patches.

The influence of PCE on the spatial distribution of these Ca–P-enriched regions is illustrated in Figure 16. In the PCE-0.0 mixture (Figure 16a), a relatively continuous fine-grained layer covers large areas of the PG surface and coexists with visible dissolution steps. The corresponding EDS spectrum (EDS-1) shows pronounced Ca and P signals, while signals from S, Si, and Al remain comparatively weak within the enriched region, indicating the formation of a continuous Ca–P-rich surface layer. Such a layer may act as a passivation-like interfacial film, partially limiting local dissolution and regulating the release of ionic species from the PG surface.

In contrast, the PCE-0.3 mixture (Figure 16b) displays a discontinuous distribution of Ca–P-enriched regions. Instead of forming an extensive covering layer, the enriched phase appears as isolated patches, accompanied by more exposed matrix surfaces and clearer dissolution-step features. The corresponding EDS spectrum (EDS-2) likewise shows dominant Ca and P peaks, confirming that these regions remain enriched in Ca and P despite their reduced spatial continuity. The disruption of a continuous Ca–P surface layer suggests that the presence of PCE modifies the interfacial chemical environment, resulting in a more heterogeneous distribution of surface phases.

This modified interfacial structure may facilitate localized dissolution and modify the nucleation environment of hydration products at the PG surface. Such behavior is consistent with the more dispersed development of AFt observed in the SEM images (Section 3.4.2) and the moderated hydration kinetics revealed by calorimetry and rheological measurements. Overall, these observations suggest that PCE influences early hydration not only through particle dispersion but also by regulating interfacial chemical heterogeneity and the formation of surface layers on PG particles.

### 3.5. Compressive Strength

**Figure 17:**
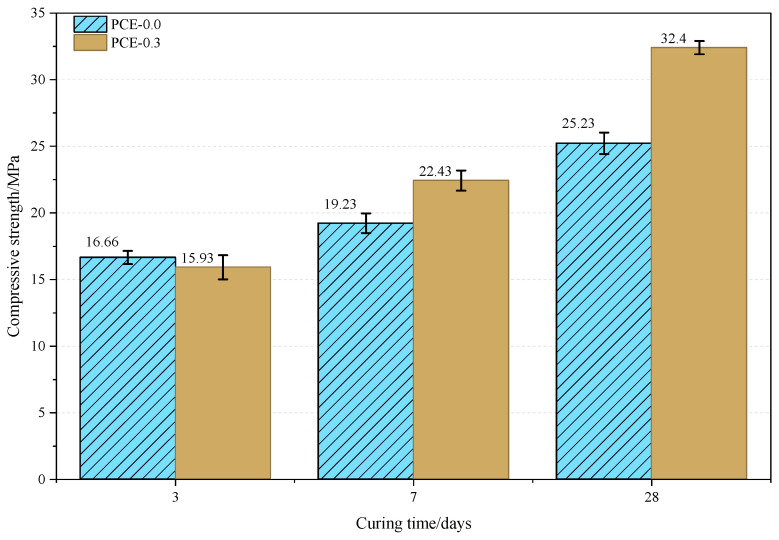
Compressive strength of PSSCM at different curing ages. The error bars represent the standard deviation of three parallel specimens.

As shown in Figure 17, the compressive strength of the PCE-0.3 mixture at 3 days (15.93 MPa) is slightly lower than that of the control mixture (16.66 MPa), indicating a modest delay in early strength development. This behavior is consistent with the delayed structural build-up and moderated early hydration kinetics discussed in Section 3.1 and Section 3.2. With increasing curing age, the PCE-0.3 mixture exhibits a higher rate of strength development. The compressive strengths at 7 and 28 days reach 22.43 MPa and 32.40 MPa, respectively, exceeding those of the control mixture (19.23 MPa and 25.23 MPa). The enhanced later-age strength may be attributed to the more uniform spatial development of hydration products and the gradual evolution of the interfacial microstructure, which promote the formation of a denser hardened matrix [43].

### 3.6. Conceptual Model of Early Evolution of PSSCM

**Figure 18:**
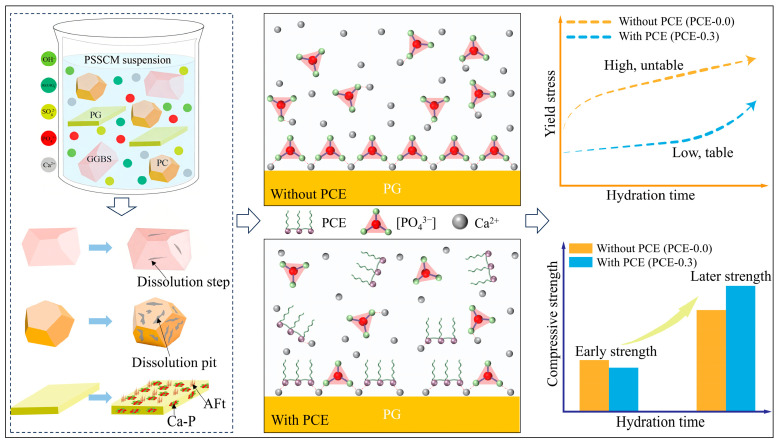
Conceptual illustration of the early evolution of PSSCM with and without PCE, showing the regulation of PG dissolution, Ca–P enrichment, and AFt nucleation, as well as their effects on rheological behavior and strength development. The blue arrows indicate the hydration process, while the red dotted lines represent the complexation between Ca^2+^ and polycarboxylate superplasticizer, as well as phosphate species.

Based on the combined results of rheological measurements, pore solution ion evolution, hydration heat analysis, and microstructural characterization (XRD, SEM, and EDS), a conceptual model describing the early evolution of PSSCM under PCE regulation is proposed, as illustrated in Figure 18.

During early hydration, PG dissolves rapidly, releasing Ca^2+^ and SO_4_^2−^ and providing preferential nucleation sites for AFt formation. Meanwhile, early dissolution of PC releases Ca^2+^ and OH^−^, creating an alkaline environment that promotes slag activation, while GGBS dissolves more slowly and mainly supplies Al species involved in AFt formation. These processes collectively establish the ionic environment governing the early precipitation of hydration products.

In the absence of PCE, impurities on PG surfaces promote the formation of relatively continuous Ca–P-enriched regions, which act as interfacial enrichment layers and provide favorable sites for localized AFt nucleation. As a result, AFt preferentially precipitates and accumulates on PG particle surfaces, forming dense and spatially concentrated AFt clusters. This localized growth leads to rapid particle bridging and early formation of a connected network, resulting in fast but less stable structural build-up and relatively high yield stress.

With the incorporation of PCE, PG dissolution becomes moderately regulated, and Ca–P enrichment changes from continuous coverage to discontinuous patches. At the same time, the adsorption of PCE on particle surfaces reduces the availability of localized nucleation sites, thereby suppressing the formation of AFt clusters at specific locations. Consequently, AFt nucleation becomes more spatially dispersed within the pore solution rather than being confined to PG surfaces. Although AFt formation still proceeds, its spatial distribution becomes more uniform, leading to continuous ion consumption but delayed formation of a percolated particle network. As a result, the structural build-up becomes slower yet more stable, accompanied by a lower and more stable yield stress. With continued hydration, the distributed AFt network gradually evolves into a more homogeneous load-bearing structure, contributing to improved later-age compressive strength.

It should be noted that this conceptual model is derived from a comprehensive interpretation of multi-scale experimental results and is intended to illustrate the relationship between interfacial reactions and macroscopic performance. It does not constitute direct evidence of specific molecular-level interaction mechanisms.

## 4. Conclusions

This study investigates the influence of PCE on the early hydration behavior and macroscopic performance development of PSSCM. Based on rheological measurements, pore solution ion evolution, hydration heat analysis, and multi-scale microstructural characterization (XRD, SEM, and EDS), the following conclusions are drawn:

1. The incorporation of PCE significantly modifies the rheological behavior of PSSCM. At dosages of 0.2–0.3 wt.%, the initial yield stress is reduced to approximately 120–300 Pa, while the structural build-up index remains relatively high (10.19–11.31), indicating improved particle dispersion without adversely affecting early structural development.
2. PG exhibits pronounced interfacial activity during early hydration, with AFt preferentially nucleating on its surface. In the absence of PCE, relatively continuous Ca–P-enriched regions are formed, leading to localized AFt precipitation. In contrast, the presence of PCE promotes more dispersed interfacial enrichment and a more homogeneous spatial distribution of AFt crystals.
3. The addition of PCE regulates early hydration kinetics by delaying gypsum consumption and AFt formation, as reflected by a shift in the onset of the acceleration stage from 11.7 h to 14.2 h. This behavior is associated with the prolonged presence of Ca^2+^ and Al(OH)_4_^−^ in the pore solution and the delayed nucleation of AFt, resulting in a reduced structural build-up rate and a more gradual progression of hydration reactions.
4. From an application-oriented perspective, incorporating PCE at 0.2–0.3 wt.% enables a balance between workability and mechanical performance, as evidenced by the reduction in yield stress and the enhancement of 28-day compressive strength (from 25.23 MPa to 32.40 MPa), despite a slight delay in early strength development. These findings provide a quantitative basis for the mix design and optimization of PSSC systems.

Overall, PCE regulates the temporal characteristics of early interfacial reactions and the spatial organization of hydration products, thereby influencing the structural evolution and macroscopic mechanical performance of PSSCM. These conclusions are derived from the integrated interpretation of multi-scale experimental results, while the detailed molecular-level interaction mechanisms still require further investigation.

## Figures and Tables

**Table 1:** Chemical compositions of the raw materials (wt.%).

Material	CaO	SiO_2_	Al_2_O_3_	MgO	SO_3_	Fe_2_O_3_	P_2_O_5_
PC	51.06	21.16	8.59	3.76	3.17	2.99	0.13
GGBS	37.48	28.35	16.64	7.24	1.3	0.25	0.02
PG	28.35	16.64	1.3	0.02	37.48	0.07	7.24

**Table 2:** Mix proportions of phosphogypsum-based supersulfated cement mortar (PSSCM) with varying PCE dosages.

Group	PG (g)	GGBS (g)	PC (g)	Sand (g)	Water (g)	PCE (g)
PCE-0.0	15	80	5	100	40	0
PCE-0.1	15	80	5	100	40	0.1
PCE-0.2	15	80	5	100	40	0.2
PCE-0.3	15	80	5	100	40	0.3
PCE-0.4	15	80	5	100	40	0.4
PCE-0.5	15	80	5	100	40	0.5
PCE-0.6	15	80	5	100	40	0.6
PCE-0.8	15	80	5	100	40	0.8

## Data Availability

The original contributions presented in this study are included in the article. Further inquiries can be directed to the corresponding author.

## Abbreviations

The following abbreviations are used in this manuscript:

PSSC	Phosphogypsum-based supersulfated cement
PSSCM	Phosphogypsum-based supersulfated cement mortar
PG	Phosphogypsum
PC	Ordinary Portland cement
GGBS	Ground granulated blast furnace slag
PCE	Polycarboxylate ether superplasticizer
$\tau_{S}$	Static yield stress
$\tau_{D}$	Dynamic yield stress
μ	Plastic viscosity
t_acc_	Characteristic time parameter

## References

[1] Gruskovnjak A., Lothenbach B., Winnefeld F., Münch B., Figi R., Ko S., Adler M., Mäder U. (2011). Quantification of hydration phases in supersulfated cements: Review and new approaches. Adv. Cem. Res..

[2] Singh M., Garg M. (2002). Calcium sulfate hemihydrate activated low heat sulfate resistant cement. Constr. Build. Mater..

[3] Novak R., Schneider W., Lang E. (2005). New knowledge regarding the supersulphated cement Slagstar. ZKG Int..

[4] Zhou B., Zhu H., Xu S., Du G., Shi S., Liu M., Xing F., Ren J. (2021). Effect of phosphogypsum on the properties of magnesium phosphate cement paste with low magnesium-to-phosphate ratio. Sci. Total Environ..

[5] Chen S., Chen J., He X., Su Y., Jin Z., Wang B. (2022). Micromicelle-mechanical coupling method for high-efficiency phosphorus removal and whiteness improvement of phosphogypsum. Constr. Build. Mater..

[6] Liu S., Fang P., Ren J., Li S. (2020). Application of lime neutralised phosphogypsum in supersulfated cement. J. Clean. Prod..

[7] Shen Y., Qian J. (2015). Utilisation of phosphogypsum for sulfate-rich belite sulfoaluminate cement production. Adv. Cem. Res..

[8] Millán-Becerro R., Pérez-López R., Cánovas C.R., Macías F., León R. (2023). Phosphogypsum weathering and implications for pollutant discharge into an estuary. J. Hydrol..

[9] Cao J., Wang Z., Ma X., Yang X., Zhang X., Pan H., Wu J., Xu M., Lin L., Zhang Y. (2022). Promoting coordinative development of phosphogypsum resources reuse through a novel integrated approach: A case study from China. J. Clean. Prod..

[10] Lam N.N., Ha-Minh C., Dao D., Benboudjema F., Derrible S., Huynh D., Tang A. (2020). A study on improvement of early-age strength of super sulfated cement using phosphogypsum. CIGOS 2019, Innovation for Sustainable Infrastructure.

[11] Liu S., Wang L., Yu B. (2019). Effect of modified phosphogypsum on the hydration properties of the phosphogypsum-based supersulfated cement. Constr. Build. Mater..

[12] Angulski da Luz C., Hooton R.D. (2015). Influence of curing temperature on the process of hydration of supersulfated cements at early age. Cem. Concr. Res..

[13] Tsioka M.M., Voudrias E. (2020). Comparison of alternative management methods for phosphogypsum waste using life cycle analysis. J. Clean. Prod..

[14] Cuadri A.A., Navarro F.J., García-Morales M., Bolívar J.P. (2014). Valorization of phosphogypsum waste as asphaltic bitumen modifier. J. Hazard. Mater..

[15] Vo M.L., Plank J. (2020). Dispersing effectiveness of a phosphated polycarboxylate in α- and β-calcium sulfate hemihydrate systems. Constr. Build. Mater..

[16] Cabrera-Luna K., Burciaga-Diaz O., Santana-Carrillo J.L., Escalante-Garcia J.I. (2025). Environmental performance of sustainable supersulfated cements based on blast furnace slag: A life cycle study. Environ. Res..

[17] Hu T.Q. (2002). Chemical Modification, Properties, and Usage of Lignin.

[18] Gazdič D., Mokrá J., Hanáček J. (2018). Influence of plasticizers on properties of anhydrite binder. Key Eng. Mater..

[19] Guan B., Ye Q., Zhang J., Lou W., Wu Z. (2010). Interaction between α-calcium sulfate hemihydrate and superplasticizer from the point of adsorption characteristics, hydration and hardening process. Cem. Concr. Res..

[20] Peng J., Qu J., Zhang J., Chen M., Wan T. (2005). Adsorption characteristics of water-reducing agents on gypsum surface and its effect on the rheology of gypsum plaster. Cem. Concr. Res..

[21] Cardoso da Silva M.R., Qi J., Ross I., Kirchheim A.P., Walkley B. (2026). Adsorption phenomena and surface interactions between superplasticisers and ground blast furnace slag and metakaolin particles in alkali solutions: Implications for low-carbon cements. Cem. Concr. Res..

[22] Chen X., Liu Y., Wu Q., Ding Y., Wang Q., Tang W., Zhu B. (2022). Study on physical and chemical characteristics of β-hemihydrate phosphogypsum. Case Stud. Constr. Mater..

[23] Plank J., Sakai E., Miao C.W., Yu C., Hong J.X. (2015). Chemical admixtures—Chemistry, applications and their impact on concrete microstructure and durability. Cem. Concr. Res..

[24] Lei L., Hirata T., Plank J. (2022). 40 years of PCE superplasticizers—History, current state-of-the-art and an outlook. Cem. Concr. Res..

[25] Liao Y., Yao J., Deng F., Li H., Wang K., Tang S. (2023). Hydration behavior and strength development of supersulfated cement prepared by calcined phosphogypsum and slaked lime. J. Build. Eng..

[26] Rubert S., Luz C., Varela M., Filho J., Hooton D. (2018). Hydration mechanisms of supersulfated cement: The role of alkali activator and calcium sulfate content. J. Therm. Anal. Calorim..

[27] Smadi M.M., Haddad R.H., Akour A.M. (1999). Potential use of phosphogypsum in concrete. Cem. Concr. Res..

[28] Jiang L., Li C., Wang C., Xu N., Chu H. (2018). Utilization of flue gas desulfurization gypsum as an activation agent for high-volume slag concrete. J. Clean. Prod..

[29] Değirmenci N. (2008). Utilization of phosphogypsum as raw and calcined material in manufacturing of building products. Constr. Build. Mater..

[30] Andrade Neto J.S., Bersch J.D., Silva T.S.M., Rodríguez E.D., Suzuki S., Kirchheim A.P. (2021). Influence of phosphogypsum purification with lime on the properties of cementitious matrices with and without plasticizer. Constr. Build. Mater..

[31] Liu S., Wang L. (2018). Investigation on strength and pore structure of supersulfated cement paste. Mater. Sci..

[32] Dong S., Yu S., Chen L., Zhuo Q., Wu F., Xie L., Liu L. (2023). Effects of pretreated phosphogypsum and granulated blast-furnace slag on the rheological properties of the paste excited by NaOH. Molecules.

[33] Gracioli B., Angulski da Luz C., Beutler C.S., Pereira Filho J.I., Frare A., Rocha J.C., Cheriaf M., Hooton R.D. (2020). Influence of the calcination temperature of phosphogypsum on the performance of supersulfated cements. Constr. Build. Mater..

[34] Xing J., Zhou Y., Peng Z., Wang J., Jin Y., Jin M. (2023). The influence of different kinds of weak acid salts on the macro-performance, microstructure, and hydration mechanism of supersulfated cement. J. Build. Eng..

[35] Wu Y., Xu F., Wu X., Jiao Y., Sun T., Li Z., Yang F., Li H., Li B., Xu J. (2024). Retardation mechanism of phosphogypsum in phosphogypsum-based excess-sulfate cement. Constr. Build. Mater..

[36] Qi H., Ma B., Tan H., Su Y., Jin Z., Li C., Liu X., Yang Q., Luo Z. (2021). Polycarboxylate superplasticizer modified by phosphate ester in side chain and its basic properties in gypsum plaster. Constr. Build. Mater..

[37] Qi H., Tang D., Ma B., Tan H., He X., Su Y. (2023). Influence of H_3_PO_4_ and H_2_PO_4_^−^ on the performance of PCE in hemihydrate gypsum pastes. Constr. Build. Mater..

[38] Qi H., He L., Tan H., He X., Yang J., Su Y., Jin Z., Zhang J. (2024). Effect of phosphate impurities on the properties of β-hemihydrate gypsum pastes plasticized by polycarboxylate superplasticizer. Mater. Today Commun..

[39] Sha S., Wang Y., Ye H. (2024). On the action mechanism of phosphate-based superplasticizers in one-part alkali-activated slag. Cem. Concr. Res..

[40] Caruso F., Mantellato S., Palacios M., Flatt R.J. (2017). ICP-OES method for the characterization of cement pore solutions and their modification by polycarboxylate-based superplasticizers. Cem. Concr. Res..

[41] Ye Q. (2021). Effect of polycarboxylate ether (PCE) superplasticizer on thixotropic structural build-up of fresh cement pastes over time. Constr. Build. Mater..

[42] Zhu W., Feng Q., Luo Q., Bai X., Chen K., Lin X. (2022). Effect of a specific PCE superplasticizer on the initial dissolution and early hydration of Portland cement. J. Build. Eng..

[43] Fang Y., Zhang X., Yan D., Lin Z., Ma X., Lai J., Liu Y., Ke Y., Chen Z., Wang Z. (2023). Study on Dispersion, Adsorption, and Hydration Effects of Polycarboxylate Superplasticizers with Different Side Chain Structures in Reference Cement and Belite Cement. Materials.

